# Non-invasive assessment of portal hypertension: Liver stiffness and beyond^☆^

**DOI:** 10.1016/j.jhepr.2024.101300

**Published:** 2024-12-11

**Authors:** Mattias Mandorfer, Juan G. Abraldes, Annalisa Berzigotti

**Affiliations:** 1 Department of Medicine III, Division of Gastroenterology and Hepatology, Medical University of Vienna, Vienna, Austria; 2 Vienna Hepatic Hemodynamic Lab, Department of Medicine III, Division of Gastroenterology and Hepatology, Medical University of Vienna, Vienna, Austria; 3 Division of Gastroenterology (Liver Unit). University of Alberta, Edmonton, Alberta, Canada; 4 Department of Visceral Surgery and Medicine, Inselspital, Bern University Hospital, University of Bern, Switzerland; 5 Department for BioMedical Research, Visceral Surgery and Medicine, University of Bern, Switzerland

**Keywords:** Advanced chronic liver disease, Cirrhosis, Varices, Decompensation, Non-invasive, Elastography

## Abstract

Portal hypertension (PH) leads to life-threatening clinical manifestations such as bleeding from gastro-oesophageal varices, ascites and its complications, and portosystemic encephalopathy. It can develop because of advanced chronic liver disease (ACLD) or due to rarer causes such as vascular liver disease. Reference standard methods to assess PH in ACLD include the measurement of hepatic venous pressure gradient and endoscopy, which have limitations due to their high resource utilisation and invasiveness. Non-invasive tests (NITs) have entered clinical practice and allow invasive procedures to be reserved for patients with indeterminate findings on NITs or for specific clinical questions. In this review, we present an update on the role of NITs, and in particular ultrasound elastography, to diagnose PH in ACLD and vascular liver disease, and to stratify the risk of liver-related events. We also provide insights into the open research questions and design of studies in this field.

**Key points**

• Non-invasive tests have been integrated into clinical practice in the setting of portal hypertension.
• The combination of imaging, laboratory tests and elastography (liver and spleen) enables classification of portal hypertension in the majority of cases of unknown origin.
• Elastography techniques have improved the sensitivity of non-invasive detection of portal hypertension in patients with compensated advanced chronic liver disease and allow invasive tests to be reserved for a minority of cases.
• Non-invasive tests allow for prognostic stratification in compensated advanced chronic liver disease.
• Non-invasive tests for portal hypertension in metabolic dysfunction-associated steatotic liver disease are currently being extensively studied and optimised.

## Introduction

Portal hypertension (PH) is a common clinical syndrome defined by an increased pressure in the portal venous system. Consequently, the pressure gradient between the portal venous system and the systemic venous circulation is increased,^1^ with the only exception being some cases of congestive hepatopathy, including Fontan-associated liver disease. The increased porto-systemic pressure gradient is the result of increased resistance to portal venous flow, which can occur at the pre-hepatic, intrahepatic and post-hepatic level,^2^ and is aggravated by the secondary increase in porto-systemic blood flow (Ohm’s law: P = R ∗ Q).

**Fig. 1:**
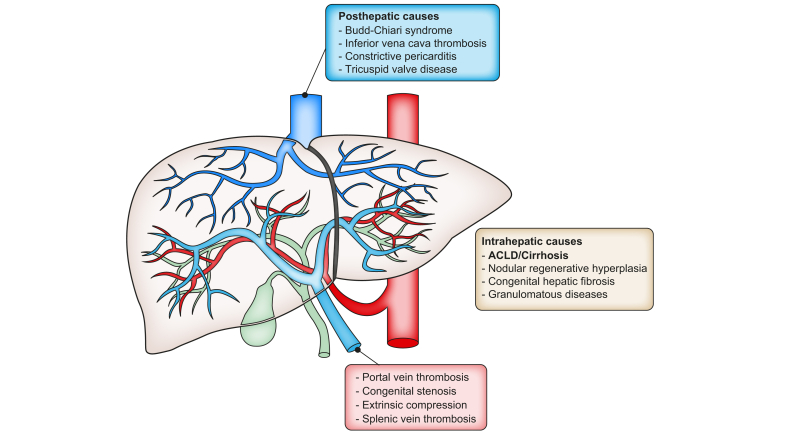
Classification of portal hypertension based on the site of increased resistance. ACLD, advanced chronic liver disease.

While pre-hepatic and post-hepatic causes of PH are comparatively rare, and mostly due to thrombotic vascular diseases (*i.e.* portal vein thrombosis and Budd-Chiari syndrome), intrahepatic PH is common, with over 80-90% of cases in the Western world being due to chronic liver diseases that lead to advanced liver fibrosis/cirrhosis, *i.e*. advanced chronic liver disease (ACLD), increasing resistance at the sinusoidal level (Fig. 1). Intrahepatic presinusoidal causes of PH are increasingly recognised, and are mostly related to portosinusoidal vascular disorder (PSVD).^3^

The measurement of hepatic venous pressure gradient (HVPG) obtained by hepatic vein catheterisation is a reference standard method to diagnose sinusoidal PH in ACLD.^1^ HVPG is considered normal up to a value of 5 mmHg, while PH is diagnosed as ‘subclinical’ for values between 6 and 9 mmHg, and as ‘clinically significant’ (CSPH) for values ≥10 mmHg. The latter term has been chosen since, above this threshold, patients are prone to develop all the clinical consequences and complications of the syndrome (development of gastro-oesophageal varices and other porto-systemic collaterals, bleeding from varices, ascites and its complications, as well as hepatic encephalopathy).^1^ CSPH may be present in around 40-60% of patients with compensated ACLD (cACLD), and even beyond the threshold of 10 mmHg, HVPG maintains a strong independent prognostic value.^4^ Nonetheless, the HVPG measurement is invasive, requires expertise to perform and interpret, and is not widely available. Gastro-oesophageal varices commonly develop in patients with CSPH; once formed, varices tend to increase in size. Large varices or varices with red signs (indicating a thin wall) are at high risk of rupture. Importantly, pharmacological and non-pharmacological strategies to lower portal pressure can reduce all the complications of PH.^5^

Non-invasive tests (NITs) able to mirror PH, and specifically to rule-out and rule-in the presence of CSPH and of gastro-oesophageal varices in patients with cACLD, have been the subject of intensive research. Besides long-standing tests such as laboratory parameters and imaging assessment, the availability of ultrasound elastography led to a major improvement in non-invasive prediction rules for PH.^6^ First, elastography identifies those with advanced liver fibrosis^6^ and/or patients at risk of liver-related events,^7^ thereby shaping the term cACLD, *i.e*. the target population for PH assessment. Second, liver stiffness has now become the backbone of non-invasive evaluation of suspected PH and holds prognostic value in this population, while spleen stiffness is increasingly entering clinical practice. In addition, novel combinations of tests unrelated to elastography have been proposed and are being investigated.

Since any test has advantages and limitations, physicians taking advantage of NITs should not only master the techniques, but also their interpretation, accounting for potential confounders related to the clinical scenarios in which the tests are used. These aspects will be discussed in this review, together with the latest developments in this field.

## Methodological insights: Diagnosis as risk prediction

Establishing a diagnosis in a patient is most useful when the diagnosis conveys prognostic information, and even more so if it has an impact on management.^8^ For example, diagnosing the presence of CSPH not only implies that the patient is at risk of decompensation but also that the patient could benefit from being treated (for example, with carvedilol/non-selective betablockers [NSBBs]). When addressing how to use diagnostic tools, traditional metrics of diagnostic performance have limited utility for decision making.^9^ As an example, sensitivity is the probability of obtaining a positive test given the presence of the disease, which is rarely a relevant clinical question in patient care. On the one hand, it requires classification of the test as positive or negative, and on the other hand, it is a backward probability because the flow of information goes in the wrong direction (from the presence of the disease/condition to the test result).

The relevant clinical question would be answered by providing a forward probability: In a given patient in a given clinical context, what would be the probability that a patient has the target condition according to the values of the diagnostic test/diagnostic model? This approach was followed, for example, in several of the manuscripts by the ANTICIPATE group, in which different risk prediction models were provided to estimate the probability of CSPH.^10^^,^^11^ Approaching the diagnostic question within a risk prediction modelling framework also allows for the combination of the given diagnostic test with additional easily available information. For example, the ANTICIPATE-NASH model to predict CSPH in patients with metabolic dysfunction-associated steatotic liver disease (MASLD) uses the values of liver stiffness measurement (LSM), platelet count, and BMI in its predictions and outperforms LSM alone in predicting CSPH and clinical outcomes.^11^^,^^12^ Models can be constructed using either traditional regression^11^^,^^12^ or with machine learning (ML) approaches.^13^ It is important to note that ML approaches require a large amount of data and are prone to overfitting.

A transparent presentation of risk prediction models that allows for easy calculation of predicted risks is important to ensure usability. Thus, full equations should be provided. Nomograms are graphical representations of the models that allow one to easily grasp the weight of each individual variable in the model and make a rough estimation of the predicted risks. Online calculators allow for more precise estimations (https://www.bcn-liverhuvh.com/resources). However, these should be viewed as medical devices, since they are intended to lead to clinical decisions, and ideally should only be made widely available once the model has been thoroughly validated. Validation involves the assessment of both discrimination and calibration. Discrimination relates to the capacity of the model to rank a group of patients and is commonly assessed with the c-statistic or AUROC. Good discrimination is especially important for models aimed at making decisions related to a group of patients (for example, ranking patients on the waiting list for transplant, or ordering patients on a waiting list for specialist referral). Calibration relates to the ability to predict absolute risks (how closely the predicted probabilities agree with the actual risk of the outcome variable).^14^ Good calibration is essential for individual decision making. For example, if – according to a utility analysis – an intervention is indicated above a certain risk threshold, getting the predicted absolute risk right (good calibration) is a pre-requisite for use of the model.^14^

Along these lines, one of the advantages of this approach is that the probabilities provided by a well calibrated model carry their own error measurements. For example, when using a model to predict CSPH and thus determine whether to treat with carvedilol/NSBBs, in a patient with a probability of CSPH of 65%, we can estimate that if we do not treat the patient, the chance of this being an error would be 65%, whereas if we decide to treat, the chance of it being an error would be 35%. Knowledge of these error rates allows for a sound utility analysis of the benefits/harms of implementing an intervention at a certain risk level and is the basis for establishing risk thresholds. Therefore, defining these thresholds and the subsequent risk strata is independent of the model-building process.^15^ The threshold set for a particular intervention or decision depends on the intervention/decision being considered. It seems natural that decision thresholds differ for interventions according to their balance of benefit/harm.

Notably, a recent review article thoroughly addressed the development and validation of risk-prediction models in hepatology.^16^

## Starting point: The compensated patient with ongoing liver injury

### Blood-based tests

Platelet count (PLT) is a very simple, and thus, broadly applied blood-based biomarker of PH. However, its isolated diagnostic performance for CSPH in patients with cACLD is insufficient for clinical decision-making (*e.g.* AUROC: 0.787^17^). PLT has been integrated into a series of widely adopted criteria and prediction models that also include ultrasound elastography-derived information (*e.g.* Baveno VII,^7^ ANTICIPATE ± NASH,^10^^,^^11^ as well as the recently published non-invasive CSPH estimated risk [NICER] model that additionally comprises spleen stiffness measurement [SSM] at 100 Hz, LSM, and BMI^18^). These criteria/models are reviewed in the dedicated ‘liver stiffness’ and ‘spleen stiffness’ sections. Moreover, ML revealed that combinations of PLT and readily available liver function tests/enzymes (*e.g*. 3P: PLT, bilirubin, and international normalised ratio as well as 5P: PLT, bilirubin, cholinesterase, gamma-glutamyltransferase, and activated partial thromboplastin time) may have some value for diagnosing CSPH in patients with cACLD.^13^ However, given their heterogenous performance in external validation cohorts and due to the increasing availability of NITs which may have a more robust performance (*i.e.*, ultrasound elastography and other more direct blood-based biomarkers of PH) it is unclear whether these models will be implemented in clinical practice.

In addition, several studies have investigated the repurposing of simple/non-patented liver fibrosis scores comprising indirect liver fibrosis markers (*e.g*. PLT) as surrogates of PH. As reviewed in detail previously,^19^^,^^20^ there was some correlation with HVPG (most likely due to the consideration of PLT), in particular, if non-ACLD or decompensated (*i.e*. easily classifiable) patients were included. However, none of the NITs is sufficiently accurate to be of clinical value beyond the detection of patients with cACLD, *i.e.* the at-risk population for CSPH and liver-related events.^19^

Similar considerations apply for patented NITs such as Fibrotest^21^ or enhanced liver fibrosis (ELF).^22^^,^^23^ The latter is increasingly used as a second-line NIT for staging liver fibrosis and comprises HA (hyaluronic acid),^24^ PIIINP (procollagen III amino-terminal peptide),^25^ and TIMP-1 (tissue inhibitor of metalloproteinase 1),^26^ *i.e*. three biomarkers of extracellular matrix remodelling that have also been individually linked to PH. In addition to liver fibrosis, as part of the structural component of intrahepatic resistance, a dynamic intrahepatic resistance component also contributes to PH, which is nowadays known to be strongly influenced by hepatic inflammation.^27^ To capture both components of intrahepatic resistance and potentially also hyperdynamic circulation, which may be related to systemic inflammation (SI) and increased portal venous flow, Sandhal *et al.*^28^ combined ELF with soluble CD163, a macrophage activation marker that had previously been linked to PH. This tended to further improve the diagnostic performance (AUROC derivation/validation 0.91/0.9),^22^ compared to ELF alone. However, this finding has to be interpreted with caution, as this comparatively small study included patients with decompensated cirrhosis, who are easier to classify with regard to CSPH.

Patients with ACLD exhibit high von Willebrand factor (VWF) antigen levels, which is attributed to endothelial dysfunction in the context of PH and bacterial translocation/SI.[29], [30], [31] Endothelial dysfunction increases the release of VWF,^32^ leading to high levels in the portal and systemic circulation.^33^ Although other VWF-related biomarkers (*e.g*. VWF activity, which is also influenced by ADAMTS-13-mediated cleavage) show a comparable correlation with HVPG, they are less commonly available and none of them exceeds VWF antigen in regard to diagnostic performance for CSPH.^32^ Important limitations of VWF antigen as a NIT for CSPH are that it should not be determined during (suspected) bacterial infections, as superimposed SI may lead to false positives; in addition, statin treatment may impact both VWF antigen and HVPG, indicating that more data on their association in this patient population are needed. All things considered, VWF antigen is a highly capable NIT for CSPH in cACLD;^34^ however, the currently available literature on this application is based on the Diagnostica Stago assay, while other widely available automated assays (*e.g.* Werfen or Siemens) seem to measure systematically lower values,^35^ thereby impeding the calibration of criteria/models that have been developed using the Diagnostica Stago assay. The inter-assay discrepancies may be explained by von Willebrand disease having been the main reason behind their development and the broad clinical implementation. Von Willebrand disease is the most common inherited bleeding disorder, of which some subtypes are accompanied by abnormally low VWF antigen values. Thus, the intended clinical use of these automated assays is the differentiation of pathologically low from normal VWF antigen values, which has implications on their linearity range. As a consequence, samples from patients with ACLD usually have to be diluted according to the manufacturer's instructions and although precision/intermediate precision is minimal with a coefficient of variation of only 3% for the Diagnostica Stago assay,^36^ it will require a thorough inter-assay/-laboratory comparison to develop a universally applicable (*i.e*. vendor-unspecific) diagnostic (and prognostic) model.

The diagnostic performance of VWF antigen for CSPH can be further improved by dividing it by PLT, which results in the VWF antigen to PLT ratio (VITRO).^37^ Since VITRO is VWF antigen-derived, it shares the aforementioned limitations. VITRO has been evaluated for several potential applications. First, the LSM/PLT-based Baveno VII criteria (see ‘*Evidence in favour of the use of liver stiffness*’ for details) result in a high proportion (∼50%) of unclassifiable patients (‘diagnostic grey zone’) and VITRO yielded an AUROC of 0.909 for diagnosing CSPH in this population. Applying cut-offs of <1.5 and ≥2.5 for ruling-out and ruling-in CSPH, respectively, the diagnostic grey zone was substantially reduced with only ∼15% of patients remaining unclassifiable.^38^ Notably, the latter group of patients had a negligible risk of hepatic decompensation, indicating that they could be treated similarly to patients without CSPH. Secondly, VITRO may be applied in a solely blood-based algorithm for diagnosing cACLD and CSPH. Evaluating a cohort comprising nearly 8,000 patients with a high pre-test probability of cACLD, FIB-4 was comparably predictive of liver-related events as LSM, with ≥1.75 being the equivalent of 10 kPa.^39^ Applying VITRO cut-offs of <1 (CSPH ruled-out) and ≥2.5 (CSPH ruled-in) to patients with FIB-4-defined cACLD resulted in a comparable diagnostic performance as the LSM/PLT-based Baveno VII criteria.

In conclusion, despite a considerable number of blood-based NITs showing a correlation with HVPG (Table 1), they are not broadly applied in the clinic except for PLT (if combined with other parameters). However, VWF and particularly VITRO score showed a robust performance in patients with cACLD, which has also been validated externally. Importantly, the discrepancies between widely available automated assays have to be resolved to extend its clinical applicability. Once this issue is resolved, the combination of blood-based tests for cACLD to identify those with advanced liver fibrosis^6^ and/or at risk of liver-related events,^7^ plus the subsequent calculation of VITRO in those with cACLD to stratify CSPH risk, may expand cACLD/CSPH diagnostics to settings where elastography is not available.^39^

**Table 1:** Summary of current blood-based and imaging NITs for evaluating PH as well as comparison with reference tests.

Test	Description	Clinical setting and applicability	Accuracy	Estimated cost
**Blood-based**				
PLT	Low PLT is associated with splenomegaly and PH	Routine liver disease assessment; PLT weakly inversely correlates with HVPG	Moderate: often used in combination with other tests to increase accuracy (see below)	EUR∗ 4
VWF	Evaluates endothelial dysfunction and is indicative of advanced liver disease, PH, and systemic inflammation	Diagnostic and prognostic marker in patients with cACLD	Lack of inter-assay/-laboratory comparison limits clinical use	EUR∗ 10
VITRO	Ratio of WWF and PLT	Diagnostic and prognostic marker in patients with cACLD	Lack of inter-assay/-laboratory comparison for VWF limits clinical use	EUR∗ 14
ELF	Serum biomarker-based test measuring extracellular matrix remodelling, *i.e*. fibrosis; useful for detecting significant/advanced fibrosis	Diagnostic test for liver fibrosis and (to a lesser degree) PH; predicts hepatic decompensation	Highly accurate second-line NIT for liver fibrosis; limited accuracy for PH	EUR∗ 173
**Imaging**				
Ultrasound plus Doppler ultrasound ± elastography	Easily accessible imaging to evaluate the liver, spleen, and adjacent vasculature and estimate flow presence and characteristics; elastography integrated into ultrasound or stand-alone (VCTE)	Diagnostic test; portal vein velocity: prognostic value; elastography (in particular, VCTE) well-established NIT for diagnosing cACLD and PH that confers prognostic information	Ultrasound varies with operator expertise and most signs have low sensitivity; combining VCTE with PLT and other information (*e.g*., NICER model) attains an AUROC of 0.9 for CSPH – highest accuracy in appropriately designed multicentre studies	Ultrasound: EUR∗ 99; USD∗∗ 130; Elastography: EUR 56-67; USD∗∗ 131
Contrast-enhanced CT scan	Cross-sectional imaging to visualise structural changes in the liver, spleen, and adjacent vasculature	Diagnostic test; liver surface nodularity reflects PH	High accuracy for detecting porto-systemic collaterals and other signs of PH	EUR∗ 391; USD∗∗ 449
MRE; GA-MRI ± MRCP	Provides detailed imaging of liver, spleen, and portal veins; useful for assessing liver stiffness and fibrosis (MRE) as well as hepatic function (GA)	Diagnostic test; can be combined with MRE; hepato-specific contrast (GA) uptake/excretion is a marker of liver function/prognostic indicator	High accuracy for detecting porto-systemic collaterals and other signs of PH	MRE: USD∗∗ 283; GA-MRI + MRCP: EUR∗ 1081
**Reference tests**				
EGD	Provides information on varices and portal hypertensive gastropathy	Diagnostic test	High accuracy for detecting porto-systemic collaterals (*i.e*., varices) and other signs of PH	EUR∗ 279; USD∗∗ 981
HVPG	Provides an accurate estimate of the porto-systemic pressure gradient in ACLD	Minimally invasive reference standard that requires considerable expertise and resources; limited by presinusoidal PH (*e.g*., PSVD)	Provides an exact measurement of the porto-systemic pressure gradient	EUR∗ 830

ACLD, advanced chronic liver disease; cACLD, compensated ACLD; CSPH, clinically significant portal hypertension; EGD, esophagogastroduodenoscopy; ELF, enhanced liver fibrosis; HCC, hepatocellular carcinoma; HVPG, hepatic venous pressure gradient; LSM, liver stiffness measurement; MRCP, magnetic resonance cholangiopancreatography; MRE, magnetic resonance elastography; NICER, non-invasive CSPH estimated risk; NITs, non-invasive tests; PH, portal hypertension; PLT, platelet count; PSVD, portosinusoidal vascular disorder; SWE, shear wave elastography; VCTE, vibration-controlled transient elastography; VITRO, VWF/PLT ratio; VWF, von Willebrand factor.

Estimates do not include associated costs (*e.g*., blood draw).

∗ Derived from the 2024 pricing of a single central European tertiary centre.

∗∗ Derived from Medicare Procedure Price Lookup. Prices shown are national averages, based on Medicare’s 2024 payments and copayments (U.S. Centers for Medicare and Medicaid Services. Procedure Price Lookup [Internet]. Baltimore (MD): U.S. Centers for Medicare and Medicaid Services; [cited 2024 Oct 26]. Available from: https://www.medicare.gov/procedure-price-lookup/).

### Imaging

Ultrasound, CT and MRI (Table 1) can identify the anatomical changes related to PH, such as dilatation of the portal venous system, increased spleen size (splenomegaly), and presence of porto-systemic collaterals. The latter are 100% specific for PH, and when they are identified on any of the imaging methods, they are sufficient to diagnose CSPH in cACLD.^6^ Spleen size maintains an independent predictive value for CSPH in compensated patients, and the parameter can be used in addition to LSM and PLT.^17^ Nonetheless, most of these signs are not sensitive, and their absence on ultrasound (and likely on the other imaging techniques) cannot be used to rule-out CSPH.^40^ On grey scale ultrasound^41^ and on CT, the liver surface nodularity (LSN) is related to the presence of cirrhosis, and correlates with PH. On CT it can be quantified using a specific software,^42^ which provides an objective parameter to estimate the presence of CSPH. In a study involving 189 patients, the AUROC of the LSN score was 0.87 ± 0.04; a LSN score >2.8 had a positive predictive value of 86% for CSPH; the prediction rule was externally validated in 89 patients, but this parameter has not entered clinical routine yet.

Doppler ultrasound signs, such as a decrease in portal vein flow velocity, increase in resistance index of the hepatic artery, splenic artery and renal arteries, and decrease in resistance index of the superior mesenteric artery, as well as flattening of the flow pattern in the hepatic veins, have all been linked to the presence of CSPH, but are not accurate enough to rule-out and rule-in this condition in compensated patients.^40^

Contrast-enhanced ultrasound transit times have been tested in the past and are reduced in patients with CSPH; however, the proportion of indeterminate cases due to overlap between patients with and without CSPH make this test unsuited to be used in clinical practice. Subharmonic pressure estimation using Sonazoid has shown promising results in a study including 45 cases^43^ with a good correlation with the HVPG (R = 0.82); a subsequent study in 125 patients confirmed that the subharmonic gradient correlates with the HVPG, but to a lesser extent (R = 0.68).^44^ A large multicentric validation study is currently ongoing (NCT05470205).

MRI has both the capability of providing anatomical imaging, and of providing data on different properties of the liver tissue (multiparametric assessment). Since gadoxetic acid uptake and excretion in liver cells is regulated by specific transporters that require hepatocyte integrity, relative liver enhancement and portal vein hyperintensity correlate with the presence of HVPG >12 mmHg, independently of other imaging features of PH.^45^ Another gadoxetic acid-enhanced MRI-based assessment, the functional liver imaging score, holds prognostic value for hepatic decompensation in cACLD; however, it has not been investigated with regard to PH.^46^ Finally, MR-based flow measurements (*i.e.* azygos blood flow and portal/hepatic artery fraction) have been tested in patients undergoing HVPG measurement and correlate with HVPG,^47^ but validation is awaited. In a pilot experience in 19 patients, spleen iron-corrected T1 relaxation time correlated with the HVPG (Spearman’s rho = 0.69), with an AUROC of 0.92 for CSPH, and performed better than liver parameters.

### Liver stiffness

Elastography techniques are based on the principle that all tissues have inherent mechanical and elastic properties that can be measured by evaluating the tissue's response to a mechanical stimulus. ‘Ultrasound elastography’ refers to a group of techniques that use ultrasound to measure the velocity of microdisplacements (shear waves) in the tissue.^48^ The use of elastography to test liver stiffness initially focused on the identification and staging of fibrosis, which significantly alters the structure of the liver. While the healthy liver is a non-stiff and compliant organ, fibrotic livers show increased stiffness, which is proportional to the amount of fibrosis.^49^

Nonetheless, liver stiffness is not only the result of fibrosis content (matrix stiffness), but rather reflects all mechanical forces acting within the organ,^48^ including hydrostatic pressure, shear stress, tensile stress, and compression stress. Interestingly, environmental mechanical properties influence cellular behaviour and act as an upstream driver of cellular phenotype, contributing to the progression of liver fibrosis by disrupting the balance of mechanosensing, ultimately sustaining and driving liver cell dysfunction.^50^ Thus, it was hypothesised that the prognostic value of LSM in ACLD might be partially ascribed to the accelerated fibrogenesis in patients with stiffer livers.

As for the methods to measure LSM, ultrasound elastography includes the first and most validated method – vibration-controlled transient elastography (VCTE: Fibroscan, Echosens, France), which is a stand-alone machine – as well as other shear wave elastographies (point- [pSWE] and 2D-SWE) which are embedded in ultrasound devices. In any of these techniques, a stimulus (either a vibration or a focused high-intensity, short-duration acoustic pulse) is transmitted to the liver by applying a probe to the skin in the right intercostal space, and the speed of the resulting displacement of the shear wave is measured by ultrasound. As for VCTE, the operator has only a monodimensional view of the area of interest, while on pSWE and 2D-SWE the area of interest is visualized in real-time.

A different method that can be used to measure liver stiffness is magnetic resonance elastography (MRE). Given the small sample size and design of the existing studies, data is insufficient to drive strong conclusions on the use of MRE for PH and we abstain from discussing this topic in detail.

### Rationale for the use of liver stiffness and its limitations

In the absence of other confounding factors, LSM in ACLD results from hepatic inflammation, liver fibrosis, and PH.^51^ Notably, both hepatic inflammation and liver fibrosis may contribute to the latter determinant of LSM via the functional and structural components of increased intrahepatic resistance. The structural component (*i.e*. liver fibrosis) has always been acknowledged as the main factor underlying increased intrahepatic resistance; additionally, the relevance of the functional (*i.e*. hepatic vascular tone) component is nowadays well-established and supported by a broad body of experimental and clinical (*e.g*. HVPG-lowering effect of nitrates and statins as well as enhanced efficacy of carvedilol *vs.* propranolol) evidence.^27^ However, the involvement of hepatic inflammation, for example, may compromise LSM’s ability to monitor HVPG, as evidenced by unusually low LSM values in some patients with ACLD who achieved a sustained virologic response (SVR) and thus resolution of hepatic inflammation, but still had CSPH.^52^^,^^53^ Moreover, long-term quantitative decreases in liver fibrosis may also contribute to low LSM values despite persistent histological cirrhosis,^54^ and probably even CSPH, as alterations in vascular structure may persist.^55^ Notably, these limitations of LSM may be mitigated by using the models specifically developed for estimating CSPH prevalence/cut-offs in patients who achieved removal/suppression of the primary aetiological factor,^7^ which will be addressed in the ‘Special considerations’ section.

Another important limitation of LSM (but also HVPG) is that it does not capture presinusoidal or prehepatic (components of) PH. These aspects are particularly relevant in patients with PSVD (presinusoidal PH) and/or splanchnic venous thrombosis (prehepatic PH), but also in cholestatic liver disease (presinusoidal component). In addition, there is also evidence for a presinusoidal component in MASH (metabolic dysfunction-associated steatohepatitis),^56^^,^^57^ though this does not seem to have major implications for the clinical utility of LSM and HVPG at this point. Whether hepatic fat content significantly impacts PH in humans, and whether it impacts the accuracy of LSM for PH in patients with MASLD/MASH remains a matter of controversy.^58^

### Evidence in favour of the use of liver stiffness

The aforementioned limitations should be taken into account, nonetheless, a large body of evidence has proven that LSM is a strong predictor of PH in patients with cACLD. Studies comparing LSM with HVPG showed a correlation between the two,^17^^,^^59^ which is strong in cases with HVPG up to 10 mmHg. Above this threshold, the correlation is weaker, likely due to the component of PH associated with increased portal blood flow, which is not fully reflected by LSM. Several meta-analyses confirmed the ability of LSM to diagnose CSPH. The most recent includes 26 studies with a total of 4,337 patients undergoing LSM by transient elastography and HVPG measurement.^60^ In this work, the area under the hierarchical summary receiver-operating characteristic curve was 0.91 (95% CI 0.88-0.93). LSM correlated with HVPG (R = 0.70; range 0.36-0.86). The optimal single cut-off for diagnosing CSPH was 22.8 kPa (95% CI 22.7-23.0), which had a sensitivity of 79% (95% CI 74-84%) and a specificity of 88% (95% CI 84-91%). Finally, both LSM >21 kPa and HVPG >10 mmHg predict the onset of decompensation in cACLD patients with similar accuracy,^61^ suggesting that NITs are as accurate as HVPG with regard to risk stratification, a finding that has been extended to the ANTICIPATE ± NASH model and the previously mentioned VITRO score.^62^

Given that LSM reflects CSPH, it is not surprising that it can also be used to determine the probability of having varices, and in particular high-risk varices (HRVs). The combination of LSM and platelet count (as well as spleen size) improves the ability of LSM in this setting.^63^ Based on the available data, the Baveno VI and Baveno VII consensus workshops suggested pragmatic rules based on these NITs to support clinical decision-making in patients with cACLD.

**Fig. 2:**
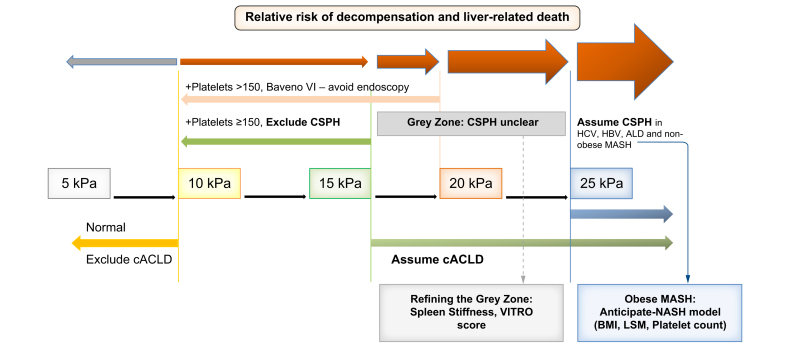
The ‘Rule of five’ supported by the Baveno VII consensus workshop.^7^ The rule, first proposed by Pons *et al.*^11^ allows for a pragmatic LSM/PLT-based risk stratification based on simple NITs to rule-in/rule-out portal hypertension and high-risk varices in patients with cACLD. ACLD, advanced chronic liver disease; cACLD, compensated ACLD; CSPH, clinically significant portal hypertension; LSM, liver stiffness measurement; MASH, metabolic dysfunction-associated steatohepatitis; PLT, platelet count; VITRO, VWF/PLT ratio; VWF, von Willebrand factor.

Fig. 2 shows the so-called rule of five proposed by Pons *et al.*^11^ and adapted by the Baveno VII consensus workshop. As shown, CSPH can be excluded in patients with LSM <15 kPa and normal PLT (*i.e*. >150 G/L), while it could be ruled-in in patients with LSM >25 kPa (except in obese MASLD, wherein BMI must also be considered; see the specific section about this population). In a large study including 1,159 patients with available follow-up, the aforementioned LSM/PLT-based Baveno VII criteria were useful to identify patients with likely CSPH, at high risk of events, and patients that likely do not have this condition, at very low risk of events.^64^ As expected, patients no longer meeting the definition of absence and not yet meeting the definition of presence of CSPH remained in a ‘diagnostic grey zone’ with intermediate risk of developing decompensation.^64^ Importantly, this intermediate risk group accounts for about half of patients observed in clinical practice, and non-invasive tools to refine diagnosis/prognosis in this setting have been established (*i.e*. VITRO and SSM, as indicated in the respective sections). Table 2 summarises the studies validating the Baveno VII criteria for CSPH to date.

**Table 2:** Performance of Baveno VII criteria for CSPH.

	Aetiology	N	CSPH	N in BVII rule-out CSPH (LSM ≤15, PLT ≥150)	N in BVII grey zone (LSM 15-24.9 kPa)	N with CSPH in the grey zone (% within the grey zone)	N in BVII rule-in CSPH (LSM ≥25)
Pons *et al.*, 2021^11^	Mixed	836	493 (59.0%)	117 (14%) CSPH: 4 NPV: 97%	362 (43.3%)	157 (43.3%)	357 (42.7%) CSPH: 332 PPV: 91%
Podrug *et al.*, 2022^101^	Mixed	76	40 (52.6%)	24 (31.6%) CSPH: 0 NPV: 100%	23 (30.3%)	15 (65.2%)	29 (38.1%) CSPH: 25 PPV: 87.1%
Jachs *et al.*, 2023^38^	Mixed, 51% viral	302	188 (62.3%)	31 (10.3%) CSPH: 0 NPV: 100%	141 (46.7%)	69 (48.9%)	130 (43%) CSPH: 119 PPV: 91.5%
Dajti *et al.*, 2022^102^	Mixed, 56% viral	195	122 (62.5%)	18 (9%) CSPH: 0 NPV: 100%	113 (58%)	62 (54.8%)	64 (33%) CSPH: 60 PPV: 93.7%
Dajti *et al.*, 2023^69^	Mixed, 77% viral	403	246 (61.0%)	48 (12%) CSPH: 0 NPV: 100%	218 (54%)	118 (54.1%)	137 (34%) CSPH: 128 PPV: 93.5%
Jindal *et al.*, 2023^103^	Mixed	626	480 (76.7%)	61 (9.7%) CSPH: 24 NV: 60.6%	277 (44.3%)	222 (80.1%)	288 (46%) CSPH: 258 PPV: 89.5%
Odriozola *et al.*, 2023^70^	Mixed, 71% MASLD	85	29 (34.1%)	27 (31.8%) CSPH: 0 NPV: 100%	54 (63.5%)	25 (46.3%)	4 (4.7%) CSPH: 4 PPV: 100%
Jachs *et al.*, 2024^104^	HDV	51	32 (62.7%)	8 (15.7%) CSPH: 0 NPV: 100%	21 (41.1%)	13 (61.9%)	22 (43.2%) CSPH: 19 PPV 86.4%
Jachs *et al.*, 2024^18^(derivation)	Mixed, 77.2% SLD, recruited from 2020-2022	202	115 (56.9%)	33 (16.3%) CSPH: 6 NPV: 81.8%	87 (43.1%)	44 (50.6%)	80 (39.6%) CSPH: 65 PPV: 81.3%
Jachs *et al.*, 2024^18^(validation)	Mixed, 68.8% SLD, recruited 2023	205	126 (61.4%)	24 (11.7%) CSPH: 1 NPV: 95.8%	111 (54.1%)	62 (55.9%)	70 (34.1%) CSPH: 63 PPV: 90%
**Total**	—	2,981	1,871 (62.8%)	391 (13%) CSPH: 35 NPV: 91.0% (95% CI 87.8-93.5%) -Likelihood ratio 0.04 (95% CI 0.03-0.06)	1,407 (47.2%)	787 (55.9%)	1,181 (39.6%) CSPH: 1,073 PPV: 90.9% (95% CI 89.1-92.4%) + Likelihood ratio 4.16 (95% CI 3.53-4.91)

ACLD, advanced chronic liver disease; cACLD, compensated ACLD; CSPH, clinically significant portal hypertension; LSM, liver stiffness measurement; MASLD, metabolic dysfunction-associated steatotic liver disease; NPV, negative predictive value; PLT, platelet count; PPV, positive predictive value; SLD, steatotic liver disease.

All studies are retrospective and included patients with cACLD based on LSM ≥10 kPa.

As for HRVs, LSM <20 kPa with normal PLT allows HRVs to be ruled-out, with <5% risk of missing this important diagnosis (Baveno VI criteria, which are now validated in all major aetiologies).^65^

Importantly, LSM correlates with the risk of hepatic decompensation and other liver-related events (onset of hepatocellular carcinoma [HCC]; risk of liver-related death), but attention should be paid to using this tool in a dynamic way. Namely, recent data have shown that beyond the initial value, LSM should be monitored during follow-up to reflect the updated status of PH. In two separate studies, either changes in LSM over time^66^ or the updated LSM value (irrespective of the initial one),^67^ were able to re-classify the risk of developing decompensation, correctly identifying patients with an improved prognosis (*e.g*. after lifestyle changes or aetiologic therapy). The importance of updating LSM over time to refine prognostication has also been confirmed in patients with alcohol-related liver disease^68^ and viral liver disease (see ‘NITs in patients with cured HCV and suppressed HBV infection’). Currently, in cACLD, a yearly measurement of LSM is recommended, but the exact interval and criteria allowing for re-classification of this risk are not yet clear, and more data are needed in this setting. In addition, natural variability in LSM can be high, and determining a level of variation sufficient to rule-out changes by chance is still a matter of debate.

### Spleen stiffness

The aforementioned limitations of LSM suggest that a more direct surrogate of portal pressure may be preferable, which prompted the study of SSM. Initially, VCTE probes/modules for LSM (50 Hz; up to 75 kPa) were repurposed and provided very encouraging results, with a correlation coefficient (R^2^ = 0.78; *i.e.* r = 0.88) and an AUROC for CSPH (0.966) that were numerically higher than for LSM. Notably, LSM also showed an outstanding performance in this study, which only included patients with compensated cirrhosis due to hepatitis C. A considerable technical failure rate (up to 24%^69^) was reported in subsequent studies, significantly decreasing the diagnostic performance of SSM when being evaluated in an intention-to-diagnose approach. The Baveno VII^7^ faculty has introduced SSM-50 Hz dual cut-offs into its consensus: while an SSM-50 Hz <21 kPa rules-out CSPH, SSM-50 Hz >50 kPa rules-in CSPH. These criteria have subsequently been confirmed by a pooled analysis; however, they resulted in a substantial diagnostic ‘grey zone’. The LSM/PLT-based Baveno VII criteria were then merged with SSM-50 Hz to reduce the proportion of unclassifiable patients from ∼50% for Baveno VII alone to 32% with the following strategy: CSPH ruled-out/-in if 2 out of 3 criteria are met (LSM ≤15 kPa, PLT >150 G/L, and/or SSM <21 kPa/LSM ≥25 kPa, PLT ≤150 G/L, and/or SSM >50 kPa). The authors also suggested a modification to further reduce the grey zone by applying a single SSM cut-off of 40 kPa instead of the 21/50 kPa dual cut-off. However, the generalisability of the mentioned SSM-50 Hz-based criteria to contemporary patients is unclear, as the proportion of viral hepatitis in the pooled analysis was as high as 77%. Moreover, given its limited technical success rate and the requirement for B-mode ultrasound to locate the spleen, SSM-50 Hz has mainly been used in the context of research and has not seen broad implementation in clinical practice.

**Fig. 3:**
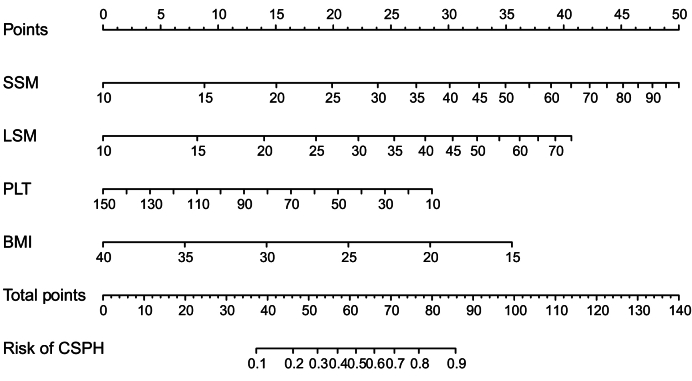
NICER model for predicting CSPH in patients with cACLD graphically illustrated as a nomogram (adapted from^18^). NICER model was developed and validated in 407 contemporary patients with cACLD, predominantly due to SLD, and outperformed ANTICIPATE ± NASH in terms of discrimination by additionally considering SSM-100 Hz. Points assigned for an individual patient’s SSM-100 Hz, LSM, PLT, and BMI are added and translate into a certain probability of CSPH. ACLD, advanced chronic liver disease; cACLD, compensated ACLD; CSPH, clinically significant portal hypertension; LSM, liver stiffness measurement; NICER, non-invasive CSPH estimated risk; PLT, platelet count; SSM, spleen stiffness measurement.

VCTE-based SSM-100 Hz using a dedicated SSM module and B-mode ultrasound integrated into the same device seems to result in a considerably lower rate of technical failure and has recently been evaluated in a Baveno Cooperation study comprising contemporary patients from 16 European centres.^18^ Among 407 patients with cACLD, with steatotic liver disease as the predominant aetiology, the technical failure rate was only 6.2%, which is in line with other SSM-100 Hz studies^70^ and compares favourably to the technical failure rate reported in the pooled analysis on SSM-50 Hz (16%).^69^ Interestingly, SSM-100 Hz did not outperform LSM as an individual test but the combination with variables considered by ANTICIPATE-NASH (*i.e*. BMI, LSM, and PLT) statistically significantly outperformed the ANTICIPATE ± NASH^10^^,^^11^ model in terms of discriminative ability (AUROC of approximately 0.9) in the derivation and validation cohorts. Notably, it was confirmed to be well-calibrated in the validation cohort, whereas ANTICIPATE ± NASH tended to underestimate risk in the medium risk category in this series of contemporary patients. Accordingly, the SSM-100 Hz-based combined model named ‘non-invasive CSPH estimated risk (NICER)’ has set a new bar for NIT-based CSPH diagnosis in cACLD and might broaden the use of SSM in clinical practice. A graphical representation of the NICER model as a nomogram is shown in Fig. 3. Finally, this multicentre study provided evidence for substituting the lower and upper SSM thresholds from 21/50 kPa to 25/55 kPa to increase diagnostic indices. Whether this finding is a consequence of differences in patient characteristics (*i.e.* contemporary cohort with steatotic liver disease as the primary aetiology) compared to previous work or the use of the 100 Hz module remains unclear.

Besides VCTE, 2D-SWE (SuperSonic Imagine [SSI]) has also been evaluated in the aforementioned pooled analysis^69^ comprising 225 patients for this specific NIT. All things considered, a similar pattern regarding the aforementioned ‘2 out of 3’ rule was observed, but the grey zone seemed to be slightly larger – which should not be overinterpreted given that this is not a head-to-head comparison. Thus, 2D-SWE (SSI) seems to be a viable alternative to SSM by VCTE, although supported by less evidence and limited by the lower availability of 2D-SWE (SSI).

In contrast, the evidence for p-SWE devices seems too limited/preliminary to draw firm conclusions regarding their diagnostic utility.^69^

Finally, MRE-based assessment of SSM has shown encouraging results in several small studies. However, these studies do not provide sufficient evidence of diagnostic utility in the main target population for this NIT, *i.e.* patients with cACLD, as they additionally included a high proportion of easily classifiable groups of patients, such as patients without ACLD or with decompensated cirrhosis.^19^ As a consequence, the latter technique is not ready for clinical use.

## Special settings

### NITs for PH in obese MASLD

**Fig. 4:**
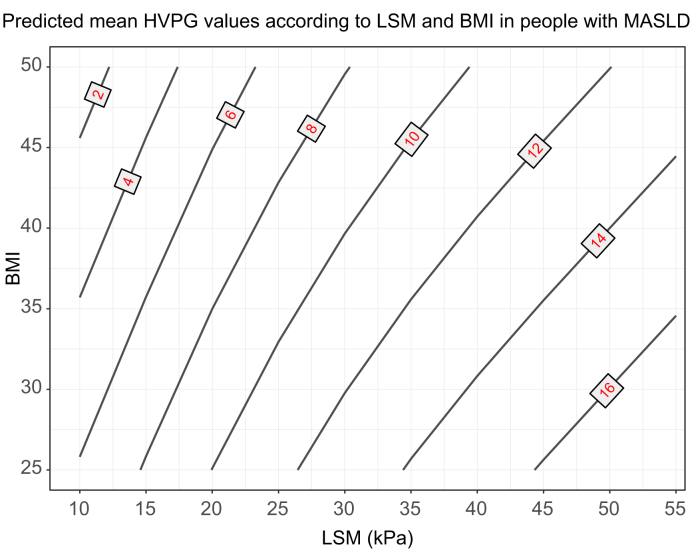
Predicted mean HVPG values according to LSM and BMI in people with MASLD. This plot shows the association between LSM and HVPG in people with MASLD (adapted from^11^). For a given value of LSM, the predicted mean HVPG is lower with higher BMIs. Figure constructed with a descriptive linear regression model developed on a sample of 234 patients with MASLD and paired measurements of LSM and HVPG. HVPG, hepatic venous pressure gradient; LSM, liver stiffness measurement; MASLD, metabolic dysfunction-associated steatotic liver disease.

In individuals with MASLD, the presence of obesity alters the association between LSM and HVPG. Indeed, for a given LSM value, an increased BMI was associated with lower HVPG values (Fig. 4). Several different findings might explain this issue, which could reflect overestimation of the degree of fibrosis by LSM in obesity.^71^ In addition, recent data suggest that in advanced MASLD cirrhosis, there might be a presinusoidal component to PH; thus, HVPG would underestimate the true portal pressure gradient, adding to uncertainties in the interpretation of the association between LSM and HVPG.^72^ It is still unknown whether this also applies to MASLD-related cACLD. Moreover, HVPG test-retest reliability seems lower in MASLD cirrhosis.^73^ Regardless of the mechanism, the impact of BMI on the association between LSM and HVPG should be considered when interpreting the decrease in LSM commonly observed with weight loss, either after lifestyle modification, drug therapy or bariatric surgery,^74^ as it may not be paralleled by a decrease in portal pressure gradient.

This differential association between LSM and HVPG results in the overestimation of the probability of CSPH with the ANTICIPATE model in patients with MASLD and obesity,^11^ which led to the development of the ANTICIPATE-NASH model. The biggest difference between the two models is the inclusion of BMI, but there are also differences in how the PLT is weighted, with more relevance given to PLT in ANTICIPATE-NASH, even at levels usually considered normal. Importantly, this model has been validated in subsequent studies, both as a predictor of CSPH^75^ and liver-related events.^12^^,^^62^^,^^76^

Based on these important considerations, the aetiology-agnostic NICER model also accounts for BMI.

### NITs in patients with cured HCV and suppressed HBV infection

Despite initial encouraging data supporting the use of LSM in the context of SVR,^77^^,^^78^ a study by Lens and colleagues^52^ raised considerable concerns regarding the diagnostic performance of LSM by VCTE for CSPH in patients who have been cured from HCV infection. However, the latter study exclusively evaluated patients who had pre-treatment CSPH as defined by HVPG, and thus, was not designed to address the question of whether LSM can differentiate between those with *vs*. without CSPH in unselected patients with cACLD who had achieved SVR. In 2022, EASL (the European Association for the Study of the Liver) noted that post-treatment NITs may be useful for risk stratification, although they should not be applied for monitoring PH.^49^ Interestingly, pooling individual patient data from all available (including the aforementioned) studies revealed that the strength of correlation between HVPG and LSM by VCTE, as well as PLT, was comparable or even stronger post-*vs.* pre-treatment.^53^ While LSM >25 kPa ruled-in CSPH (prompting the initiation/continuation of carvedilol/NSBB therapy), those with LSM <12 kPa and normal PLT had negligible risk. Of note, the consideration of PLT served as a safety net in this recommendation, since CSPH may be occasionally present despite low LSM, as highlighted by Lens *et al.*^52^ – however, these patients usually have decreased PLT. In patients meeting this composite criterium, the prevalence of CSPH was negligible (0.3%) and there were no hepatic decompensation events even during long-term follow-up, excluding patients who developed HCC.^53^^,^^79^ Thus, those who show consistent improvements in LSM to <12 kPa, as well as normal PLT, and who do not have co-factors, may be discharged from PH surveillance according to the Baveno VII consensus statement,^7^ which was adopted by the respective EASL position paper,^80^ while HCC surveillance should be continued until recommendations regarding risk-based strategies become available.

Notably, there is no data on NITs for PH (as assessed by HVPG) in patients with suppressed HBV infection; however, it seems reasonable to use the same criteria as for cured HCV. The latter consideration may also apply to patients with suppressed HBV/HDV coinfection, since in HBV/HDV-infected patients who were mostly HDV RNA positive, NITs performed similarly to other aetiologies.^81^

Finally, the Baveno VII faculty also recommended that NITs for (CS)PH may be reassessed on a yearly basis in patients with cACLD and defined a clinically meaningful change in LSM as any decrease to <10 kPa, or to 10-20 kPa with a relative decrease >20%. Despite being supported by little published data at the time of Baveno VII, the prognostic utility of these criteria has subsequently been substantiated by a study in patients with alcohol-related liver disease.^82^ However, a recent analysis in 2,335 patients with cACLD who had achieved HCV cure dissected these criteria and reported that the absolute post-treatment LSM is the key determinant of risk, while the consideration of the 20%-decrease to 10-20 kPa does not add clinically meaningful information,^83^ which was also independently reported in a mixed aetiology cohort.^67^ As a consequence, these criteria are expected to be refined/simplified by Baveno VIII.

Table 3 summarises the clinical context of use of LSM and SSM in the aforementioned scenarios.

**Table 3:** Clinical use of liver and spleen elastography in the context of diagnosing/staging PH.

Clinical situation in compensated patients with liver disease	Liver stiffness	Spleen stiffness	Explanation
Clinical, laboratory, or imaging findings of PH; unclear origin of the syndrome	Yes	Yes	Rule-out/rule-in ACLD; mismatch between LSM (<10 kPa) and specific/unspecific clinical/laboratory/imaging evidence of CSPH or SSM (>40 kPa) suggests PSVD or undiagnosed portal vein or splenic/mesenteric vein thrombosis
cACLD, PH status not yet known	Yes	Yes	LSM >25 kPa and SSM >50 kPa suggest CSPH LSM <15 kPa with normal PLT rules-out CSPH ANTICIPATE ± NASH and NICER model to estimate CSPH risk Baveno VI criteria: LSM <20 kPa and PLT>150 G/L rule-out high-risk varices
cACLD due to viral hepatitis achieving SVR (HCV), likely also applicable to viral suppression (HBV)	Yes	unclear	LSM <12 kPa as well as normal PLT in patients without co-factors may identify those who can be discharged from PH surveillance
Compensated ACLD ± PH	Yes	Yes	Prognostic value of LSM and SSM, and their changes/current value on follow-up SSM changes correlated with HVPG changes in two studies^105^^,^^106^
Patients undergoing TIPS	Likely yes	Yes	LSM and SSM decrease after TIPS; increases in LSM suggest hepatic inflammation; SSM decreases after TIPS;^107^ their increase in the follow-up might indicate TIPS dysfunction^108^

ACLD, advanced chronic liver disease; cACLD, compensated ACLD; CSPH, clinically significant portal hypertension; HVPG, hepatic venous pressure gradient; LSM, liver stiffness measurement; NICER, non-invasive CSPH estimated risk; PH, portal hypertension; PLT, platelet count; SSM, spleen stiffness measurement; VCTE, vibration-controlled transient elastography.

The reported values refer to VCTE, which accounts for the vast majority of available studies.

### NITs for PH in HCC

CSPH is a major risk factor for postoperative liver failure and decompensation after resection of HCC in patients with cACLD and Child-Pugh stage A.^84^,^85^ Presence of varices or PLT <100 G/L are inaccurate to identify CSPH in the context of HCC.^85^ LSM has been tested in a prospective study and the use of two previously established cut-offs (<13.6 kPa to rule-out and >21 kPa to rule-in CSPH) can correctly classify the majority of patients.^86^ However, in a retrospective study including 185 patients (46% Barcelona Clinic Liver Cancer [BCLC]-0/A; 26% BCLC-C; HRVs in 23%; CSPH in 42% of the subgroup of 60 patients with information on HVPG) the specificity of LSM ≥25 kPa to rule-in CSPH was only 48% and favourable Baveno VI criteria missed 11% of HRVs in BCLC 0/A.^87^ As expected, in patients with tumoral portal vein invasion, the discriminative ability of LSM was even lower, underlining an insufficient accuracy of the standard criteria for ruling-out HRV based on NITs in this population.

Data on SSM are still scarce in the setting of HCC. In a recent study performed in 219 patients with HBV-related cACLD and HCC (49% BCLC A, 38% BCLC C; HRVs in 29%) the Baveno VI criteria safely avoided 27.4% of esophagogastroduodenoscopies (EGDs) with a HRV missing rate of 3.2%. SSM-100 Hz on VCTE performed better, with a SSM ≤40 kPa enabling 47.5% of EGDs to be avoided, with a HRV missing rate of <5%.^88^

Data in other aetiologies are missing; however, in the view of the authors, patients with HCC should be evaluated using the reference standard methods whenever the presence of CSPH/varices could impact clinical decision-making.

Finally, a recent study suggests that in the setting of compensated cirrhosis and unresectable HCC, the combination of spleen volume, HCC size, BMI, portal vein diameter and serum total bilirubin might help predict the presence or absence of HRVs.^89^

PH is associated with worse outcomes in other HCC clinical scenarios (*e.g.* locoregional therapy; systemic therapy,^90^;^91^ and an accurate NIT or combination of NITs for identifying CSPH is an unmet need in this field.

Nonetheless, NITs including LSM and SSM, as well as indocyanine green clearance and VWF,^92^ hold prognostic information regarding the onset of post-hepatectomy liver failure and decompensation, and HCC recurrence, and might be used to enrich clinical assessment in this difficult population. In a cohort of 163 patients with resectable HCC, VWF showed a good discriminative accuracy to diagnose CSPH (AUROC of 0.824),^92^ and validation studies using this biomarker are awaited.

### NITs for PH in vascular liver disease

**Fig. 5:**
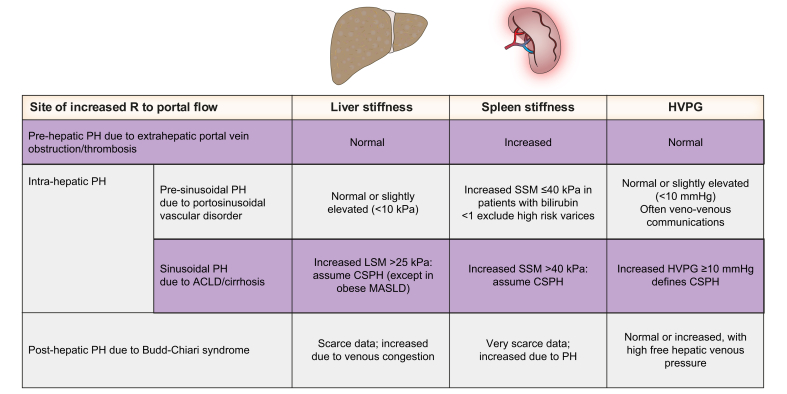
Main findings of liver and spleen stiffness measurement using transient elastography in patients with portal hypertension of different aetiologies. HVPG, hepatic venous pressure gradient; LSM, liver stiffness measurement; MASLD, metabolic dysfunction-associated steatotic liver disease; PH, portal hypertension; SSM, spleen stiffness measurement.

In patients with PH of unknown origin, NITs are useful to identify the most probable cause. Ultrasound assessment (or in second-line contrast-enhanced CT or MRI) is sensitive in the identification of thrombotic liver disease. In patients with portal vein thrombosis (PVT), LSM is helpful in differentiating cirrhotic from non-cirrhotic underlying diseases. Fig. 5 summarises the main findings of liver and spleen stiffness measurement using transient elastography in patients with portal hypertension of different aetiologies. In patients with non-cirrhotic obstructive PVT, SSM is elevated, and values are higher in patients who had already bled from varices.^93^ Based on this data, SSM could be a seen as a monitoring tool to follow-up PH in patients with PVT, but data to support this hypothesis are still missing.^94^ In Budd-Chiari syndrome, LSM is increased due to congestion, and it has been shown to decrease after transjugular intrahepatic portosystemic shunt (TIPS) placement;^95^ data on SSM are still very scarce in this field. In the authors’ experience (unpublished), a new ≥20% increase in LSM in the follow-up after TIPS reveals congestion due to TIPS dysfunction, and should prompt invasive assessment of the portal pressure gradient irrespective of Doppler findings.

Porto-sinusoidal vascular disorder (PSVD) is more difficult to differentiate from cirrhosis based on imaging.^96^ Usually, patients with PSVD present with evident clinical and laboratory signs of PH but completely preserved liver function; this mismatch can raise the suspicion of PSVD. A LSM <10 kPa, which is below ruling-out cACLD, reinforces this suspicion. The SSM to LSM ratio can be used to confirm the gap between the two, which is larger in PSVD than in patients with cirrhosis and a similar degree of PH.^97^;^98^ The diagnosis of PSVD is still based on liver biopsy, but beyond diagnosis, a recent study performed in 309 patients with PSVD (divided into a discovery cohort and a validation cohort) has shown that SSM is useful to rule-out HRVs in this vascular liver disease.^99^ Namely, SSM-VCTE ≤40 kPa combined with serum bilirubin <1 mg/dl had a sensitivity of 96% to rule-out HRVs and the use of this prediction rule could spare 38% of screening EGDs, with 4% of HRVs missed, and a 95% negative predictive value.

## Conclusions

LSM and NITs more generally have gained momentum in the management of PH. As outlined in this review, they should no longer be seen as merely diagnostic tools, but more as prognostic tools. In addition to their classical use based on a single measurement, data are increasingly showing an advantage of assessing values over the course of disease. Given the recently developed concepts of recompensation and regression of liver fibrosis, changes in NITs might become part of future definitions in these settings and will need to be validated in prospective cohorts specifically investigating these outcomes. The pragmatic use of NITs in patients with PH has been proposed by the Baveno VII consensus workshop, which suggested to start carvedilol/NSBBs on diagnosis of CSPH to prevent decompensation.^7^ Models such as ANTICIPATE ± NASH or NICER allow for the estimation of CSPH risk in individual patients, thereby providing the opportunity to weigh the probability of adequate treatment initiation *vs*. missing a diagnosis of CSPH and an opportunity for preventing hepatic decompensation. While the pragmatic use of NSBBs based on the Baveno VI criteria for HRVs has been proven cost-effective,^100^ cost-effectiveness analyses of starting NSBBs based on the Baveno VII criteria are not available yet and will be the subject of future studies. Finally, low LSM in the context of high SSM or evidence of CSPH should raise the suspicion of presinusoidal (PSVD) or pre-hepatic (PVT or splanchnic vein thrombosis) PH.

## Abbreviations

ACLD, advanced chronic liver disease; cACLD, compensated ACLD; CSPH, clinically significant portal hypertension; EGD, esophagogastroduodenoscopy; ELF, enhanced liver fibrosis; HCC, hepatocellular carcinoma; HRVs, high-risk varices; HVPG, hepatic venous pressure gradient; LSM, liver stiffness measurement; LSN, liver surface nodularity; MASLD, metabolic dysfunction-associated steatotic liver disease; ML, machine learning; MRE, magnetic resonance elastography; NICER, non-invasive CSPH estimated risk; NITs, non-invasive tests; NSBBs, non-selective betablockers; PH, portal hypertension; PLT, platelet count; PSVD, portosinusoidal vascular disorder; PVT, portal vein thrombosis; SI, systemic inflammation; SSM, spleen stiffness measurement; SSI, SuperSonic Imagine; SVR, sustained virologic response; SWE, shear wave elastography; TIPS, transjugular intrahepatic portosystemic shunt; VCTE, vibration-controlled transient elastography; VITRO, VWF/PLT ratio; VWF, von Willebrand factor.

## Financial support

MM’s work is supported the Clinical Research Group Mechanisms in Portal Hypertension (MOTION), a project funded by the Clinical Research Groups Programme of the Ludwig Boltzmann Gesellschaft (grant number LBG_KFG_22_32) with funds from the Fonds Zukunft Österreich. AB’s work is supported by a grant from the Swiss National Science Foundation (SNSF 320030-227778) and by a grant from the National Institutes of Health (NIH 2R01DK098526-05A1).

## Authors' contributions

MM, JGA and AB conducted the literature review, and drafted the manuscript. AB conceptualized the review structure. All authors provided critical revisions and approved the final version of the manuscript.

## Conflict of interest

MM served as a speaker and/or consultant and/or advisory board member for AbbVie, Echosens, Gilead, Ipsen, Takeda, and W. L. Gore & Associates and received travel support from AbbVie and Gilead. JGA consulting for 89bio, Agomab, Novo Nordisk, Boehringer Ingelheim, AstraZeneca, Terumo, Boston Pharmaceuticals. Grant support: Salix, Gilead, Cook. AB was consultant to Boehringer-Ingelheim and received speakers’ fees from GE Healthcare and Hologic.

Please refer to the accompanying ICMJE disclosure forms for further details.

## References

[1] Bosch J., Abraldes J.G., Berzigotti A. (2009). The clinical use of HVPG measurements in chronic liver disease. Nat Rev Gastroenterol Hepatol.

[2] Berzigotti A., Seijo S., Reverter E. (2013). Assessing portal hypertension in liver diseases. Expert Rev Gastroenterol Hepatol.

[3] De Gottardi A., Rautou P.E., Schouten J. (2019). Porto-sinusoidal vascular disease: proposal and description of a novel entity. Lancet Gastroenterol Hepatol.

[4] Gluud C., Brok J., Gong Y. (2007). Hepatology may have problems with putative surrogate outcome measures. J Hepatol.

[5] Villanueva C., Albillos A., Genesca J. (2019). Beta blockers to prevent decompensation of cirrhosis in patients with clinically significant portal hypertension (PREDESCI): a randomised, double-blind, placebo-controlled, multicentre trial. Lancet.

[6] de Franchis R., VI Faculty Baveno (2015). Expanding consensus in portal hypertension: report of the Baveno VI Consensus Workshop: stratifying risk and individualizing care for portal hypertension. J Hepatol.

[7] de Franchis R., Bosch J., Garcia-Tsao G. (2022). Baveno VII - renewing consensus in portal hypertension. J Hepatol.

[8] Hemingway H., Croft P., Perel P. (2013). Prognosis research strategy (PROGRESS) 1: a framework for researching clinical outcomes. Bmj.

[9] Harrel F.E. (2017). Clinicians’ misunderstanding of probabilities makes them like backwards probabilities such as sensitivity, specificity, and type I error [internet]. Nashville (TN): Harrel FE.

[10] Abraldes J.G., Bureau C., Stefanescu H. (2016). Noninvasive tools and risk of clinically significant portal hypertension and varices in compensated cirrhosis: the "Anticipate" study. Hepatology.

[11] Pons M., Augustin S., Scheiner B. (2021). Noninvasive diagnosis of portal hypertension in patients with compensated advanced chronic liver disease. Am J Gastroenterol.

[12] Pons M., Rivera-Esteban J., Ma M.M. (2024). Point-of-Care noninvasive prediction of liver-related events in patients with nonalcoholic fatty liver disease. Clin Gastroenterol Hepatol : official Clin Pract J Am Gastroenterological Assoc.

[13] Reinis J., Petrenko O., Simbrunner B. (2023). Assessment of portal hypertension severity using machine learning models in patients with compensated cirrhosis. J Hepatol.

[14] Altman D.G., Vergouwe Y., Royston P. (2009). Prognosis and prognostic research: validating a prognostic model. Bmj.

[15] Wynants L., van Smeden M., McLernon D.J. (2019). Three myths about risk thresholds for prediction models. BMC Med.

[16] Strandberg R., Jepsen P., Hagstrom H. (2024). Developing and validating clinical prediction models in hepatology - an overview for clinicians. J Hepatol.

[17] Berzigotti A., Seijo S., Arena U. (2013). Elastography, spleen size, and platelet count identify portal hypertension in patients with compensated cirrhosis. Gastroenterology.

[18] Jachs M., Odriozola A., Turon F. (2024 Dec). Spleen stiffness measurement by vibration-controlled transient elastography at 100 Hz for non-invasive predicted diagnosis of clinically significant portal hypertension in patients with compensated advanced chronic liver disease: a modelling study. Lancet Gastroenterol Hepatol.

[19] Mandorfer M., Hernandez-Gea V., Garcia-Pagan J.C. (2020). Noninvasive diagnostics for portal hypertension: a comprehensive review. Semin Liver Dis.

[20] Qi X., Berzigotti A., Cardenas A. (2018). Emerging non-invasive approaches for diagnosis and monitoring of portal hypertension. Lancet Gastroenterol Hepatol.

[21] Thabut D., Imbert-Bismut F., Cazals-Hatem D. (2007). Relationship between the Fibrotest and portal hypertension in patients with liver disease. Aliment Pharmacol Ther.

[22] Sandahl T.D., McGrail R., Moller H.J. (2016). The macrophage activation marker sCD163 combined with markers of the Enhanced Liver Fibrosis (ELF) score predicts clinically significant portal hypertension in patients with cirrhosis. Aliment Pharmacol Ther.

[23] Simbrunner B., Marculescu R., Scheiner B. (2020). Non-invasive detection of portal hypertension by enhanced liver fibrosis score in patients with different aetiologies of advanced chronic liver disease. Liver Int : official J Int Assoc Study Liver.

[24] Kropf J., Gressner A.M., Tittor W. (1991). Logistic-regression model for assessing portal hypertension by measuring hyaluronic acid (hyaluronan) and laminin in serum. Clin Chem.

[25] Gressner A.M., Tittor W., Negwer A. (1986). Serum concentrations of laminin and aminoterminal propeptide of type III procollagen in relation to the portal venous pressure of fibrotic liver diseases. Clin Chim Acta.

[26] Busk T.M., Bendtsen F., Nielsen H.J. (2014). TIMP-1 in patients with cirrhosis: relation to liver dysfunction, portal hypertension, and hemodynamic changes. Scand J Gastroenterol.

[27] Bosch J., Groszmann R.J., Shah V.H. (2015). Evolution in the understanding of the pathophysiological basis of portal hypertension: how changes in paradigm are leading to successful new treatments. J Hepatol.

[28] Gronbaek H., Sandahl T.D., Mortensen C. (2012). Soluble CD163, a marker of Kupffer cell activation, is related to portal hypertension in patients with liver cirrhosis. Aliment Pharmacol Ther.

[29] Ferro D., Quintarelli C., Lattuada A. (1996). High plasma levels of von Willebrand factor as a marker of endothelial perturbation in cirrhosis: relationship to endotoxemia. Hepatology.

[30] La Mura V., Reverter J.C., Flores-Arroyo A. (2011). Von Willebrand factor levels predict clinical outcome in patients with cirrhosis and portal hypertension. Gut.

[31] Mandorfer M., Schwabl P., Paternostro R. (2018). Von Willebrand factor indicates bacterial translocation, inflammation, and procoagulant imbalance and predicts complications independently of portal hypertension severity. Aliment Pharmacol Ther.

[32] Simbrunner B., Villesen I.F., Scheiner B. (2023). Von Willebrand factor processing in patients with advanced chronic liver disease and its relation to portal hypertension and clinical outcome. Hepatol Int.

[33] Driever E.G., Magaz M., Adelmeijer J. (2022). The portal vein in patients with cirrhosis is not an excessively inflammatory or hypercoagulable vascular bed, a prospective cohort study. J Thromb Haemost.

[34] Ferlitsch M., Reiberger T., Hoke M. (2012). von Willebrand factor as new noninvasive predictor of portal hypertension, decompensation and mortality in patients with liver cirrhosis. Hepatology.

[35] Dominik N., Scheiner B., Zanetto A. (2024 Jun). Von Willebrand factor for outcome prediction within different clinical stages of advanced chronic liver disease. Aliment Pharmacol Ther.

[36] Jachs M., Hartl L., Simbrunner B. (2022). Decreasing von Willebrand Factor Levels Upon Nonselective Beta Blocker Therapy Indicate a Decreased Risk of Further Decompensation, Acute-on-chronic Liver Failure, and Death. Clin Gastroenterol Hepatol : official Clin Pract J Am Gastroenterological Assoc.

[37] Hametner S., Ferlitsch A., Ferlitsch M. (2016). The VITRO score (von Willebrand factor antigen/thrombocyte ratio) as a new marker for clinically significant portal hypertension in comparison to other non-invasive parameters of fibrosis including ELF test. PLoS ONE.

[38] Jachs M., Hartl L., Simbrunner B. (2023). The sequential application of Baveno VII criteria and VITRO score improves diagnosis of clinically significant portal hypertension. Clin Gastroenterol Hepatol : official Clin Pract J Am Gastroenterological Assoc.

[39] Semmler G., Hartl L., Mendoza Y.P. (2024 Oct 1). Simple blood tests to diagnose compensated advanced chronic liver disease and stratify the risk of clinically significant portal hypertension. Hepatology.

[40] Berzigotti A., Piscaglia F., Education E., Professional Standards C. (2012). Ultrasound in portal hypertension--part 2--and EFSUMB recommendations for the performance and reporting of ultrasound examinations in portal hypertension. Ultraschall Med.

[41] Berzigotti A., Abraldes J.G., Tandon P. (2010). Ultrasonographic evaluation of liver surface and transient elastography in clinically doubtful cirrhosis. J Hepatol.

[42] Sartoris R., Rautou P.E., Elkrief L. (2018). Quantification of liver surface nodularity at CT: utility for detection of portal hypertension. Radiology.

[43] Eisenbrey J.R., Dave J.K., Halldorsdottir V.G. (2013). Chronic liver disease: noninvasive subharmonic aided pressure estimation of hepatic venous pressure gradient. Radiology.

[44] Gupta I., Eisenbrey J.R., Machado P. (2021). Diagnosing portal hypertension with noninvasive subharmonic pressure estimates from a US contrast agent. Radiology.

[45] Asenbaum U., Ba-Ssalamah A., Mandorfer M. (2017). Effects of portal hypertension on gadoxetic acid-enhanced liver magnetic resonance: diagnostic and prognostic implications. Invest Radiol.

[46] Bastati N., Beer L., Mandorfer M. (2020). Does the functional liver imaging score derived from gadoxetic acid-enhanced MRI predict outcomes in chronic liver disease?. Radiology.

[47] Palaniyappan N., Cox E., Bradley C. (2016). Non-invasive assessment of portal hypertension using quantitative magnetic resonance imaging. J Hepatol.

[48] Dietrich C.F., Bamber J., Berzigotti A. (2017). EFSUMB guidelines and recommendations on the clinical use of liver ultrasound elastography, update 2017 (long version). Ultraschall Med.

[49] European Association for the Study of the Liver (2021). EASL Clinical Practice Guidelines on non-invasive tests for evaluation of liver disease severity and prognosis - 2021 update. J Hepatol.

[50] Felli E., Selicean S., Guixe-Muntet S. (2023). Mechanobiology of portal hypertension. JHEP Rep.

[51] Berzigotti A. (2017). Non-invasive evaluation of portal hypertension using ultrasound elastography. J Hepatol.

[52] Lens S., Alvarado-Tapias E., Marino Z. (2017). Effects of all-oral anti-viral therapy on HVPG and systemic hemodynamics in patients with hepatitis C virus-associated cirrhosis. Gastroenterology.

[53] Semmler G., Lens S., Meyer E.L. (2022). Non-invasive tests for clinically significant portal hypertension after HCV cure. J Hepatol.

[54] Broquetas T., Herruzo-Pino P., Marino Z. (2021). Elastography is unable to exclude cirrhosis after sustained virological response in HCV-infected patients with advanced chronic liver disease. Liver Int : official J Int Assoc Study Liver.

[55] Theise N.D., Jia J., Sun Y. (2018). Progression and regression of fibrosis in viral hepatitis in the treatment era: the Beijing classification. Mod Pathol.

[56] Ferrusquia-Acosta J., Bassegoda O., Turco L. (2021). Agreement between wedged hepatic venous pressure and portal pressure in non-alcoholic steatohepatitis-related cirrhosis. J Hepatol.

[57] Bassegoda O., Olivas P., Turco L. (2022). Decompensation in advanced nonalcoholic fatty liver disease may occur at lower hepatic venous pressure gradient levels than in patients with viral disease. Clin Gastroenterol Hepatol : official Clin Pract J Am Gastroenterological Assoc.

[58] Baffy G., Bosch J. (2022). Overlooked subclinical portal hypertension in non-cirrhotic NAFLD: is it real and how to measure it?. J Hepatol.

[59] Vizzutti F., Arena U., Romanelli R.G. (2007). Liver stiffness measurement predicts severe portal hypertension in patients with HCV-related cirrhosis. Hepatology.

[60] Kumar A., Maruyama H., Arora A. (2022). Diagnostic accuracy of transient elastography in diagnosing clinically significant portal hypertension in patients with chronic liver disease: a systematic review and meta-analysis. J Med Ultrason.

[61] Robic M.A., Procopet B., Metivier S., Peron J.M., Selves J., Vinel J.P. (2011). Liver stiffness accurately predicts portal hypertension related complications in patients with chronic liver disease: a prospective study. J Hepatol.

[62] Jachs M., Hartl L., Simbrunner B. (2024). Prognostic performance of non-invasive tests for portal hypertension is comparable to that of hepatic venous pressure gradient. J Hepatol.

[63] Manatsathit W., Samant H., Kapur S. (2018). Accuracy of liver stiffness, spleen stiffness, and LS-spleen diameter to platelet ratio score in detection of esophageal varices: systemic review and meta-analysis. J Gastroenterol Hepatol.

[64] Wong Y.J., Zhaojin C., Tosetti G. (2023). Baveno-VII criteria to predict decompensation and initiate non-selective beta-blocker in compensated advanced chronic liver disease patients. Clin Mol Hepatol.

[65] Bai W., Abraldes J.G. (2022). Noninvasive assessment oesophageal varices: impact of the Baveno VI criteria. Curr Opin Gastroenterol.

[66] Semmler G., Yang Z., Fritz L. (2023). Dynamics in liver stiffness measurements predict outcomes in advanced chronic liver disease. Gastroenterology.

[67] Wong Y.J., Chen V.L., Abdulhamid A. (2025 Feb 1). Comparing serial and current liver stiffness measurements to predict decompensation in compensated advanced chronic liver disease patients. Hepatology.

[68] Thorhauge K.H., Semmler G., Johansen S. (2024). Using liver stiffness to predict and monitor the risk of decompensation and mortality in patients with alcohol-related liver disease. J Hepatol.

[69] Dajti E., Ravaioli F., Zykus R. (2023). Accuracy of spleen stiffness measurement for the diagnosis of clinically significant portal hypertension in patients with compensated advanced chronic liver disease: a systematic review and individual patient data meta-analysis. Lancet Gastroenterol Hepatol.

[70] Odriozola A., Puente A., Cuadrado A. (2023). High accuracy of spleen stiffness measurement in diagnosing clinically significant portal hypertension in metabolic-associated fatty liver disease. Liver Int.

[71] Wong V.W., Irles M., Wong G.L. (2019). Unified interpretation of liver stiffness measurement by M and XL probes in non-alcoholic fatty liver disease. Gut.

[72] Ferrusquia-Acosta J., Bassegoda O., Turco L. (2021 Apr). Agreement between wedged hepatic venous pressure and portal pressure in non-alcoholic steatohepatitis-related cirrhosis. J Hepatol.

[73] Bai W., Al-Karaghouli M., Stach J. (2021). Test-retest reliability and consistency of HVPG and impact on trial design: a study in 289 patients from 20 randomized controlled trials. Hepatology.

[74] Nixdorf L., Hartl L., Strohl S. (2024). Rapid improvement of hepatic steatosis and liver stiffness after metabolic/bariatric surgery: a prospective study. Sci Rep.

[75] Rabiee A., Deng Y., Ciarleglio M. (2022). Noninvasive predictors of clinically significant portal hypertension in NASH cirrhosis: validation of ANTICIPATE models and development of a lab-based model. Hepatol Commun.

[76] Pennisi G., Enea M., Vigano M. (2023). Oesophageal varices predict complications in compensated advanced non-alcoholic fatty liver disease. JHEP Rep.

[77] Mandorfer M., Kozbial K., Schwabl P. (2016). Sustained virologic response to interferon-free therapies ameliorates HCV-induced portal hypertension. J Hepatol.

[78] Mandorfer M., Kozbial K., Schwabl P. (2020). Changes in hepatic venous pressure gradient predict hepatic decompensation in patients who achieved sustained virologic response to interferon-free therapy. Hepatology.

[79] Semmler G., Alonso Lopez S., Pons M. (2025 Feb 1). Long-term outcome and risk stratification in compensated advanced chronic liver disease after HCV-cure. Hepatology.

[80] Reiberger T., Lens S., Cabibbo G. (2024). EASL position paper on clinical follow-up after HCV cure. J Hepatol.

[81] Jachs M., Sandmann L., Hartl L. (2024 Aug). Validation of Baveno VII criteria and other non-invasive diagnostic algorithms for clinically significant portal hypertension in hepatitis delta. J Hepatol.

[82] Thorhauge K.H., Semmler G., Johansen S. (2024 Jul). Using liver stiffness to predict and monitor the risk of decompensation and mortality in patients with alcohol-related liver disease. J Hepatol.

[83] Semmler G., Alonso Lopez S., Pons M. (2024 Jul). Post-treatment LSM rather than change during treatment predicts decompensation in patients with cACLD after HCV cure. J Hepatol.

[84] Bruix J., Castells A., Bosch J. (1996). Surgical resection of hepatocellular carcinoma in cirrhotic patients: prognostic value of preoperative portal pressure. Gastroenterology.

[85] Berzigotti A., Reig M., Abraldes J.G. (2015). Portal hypertension and the outcome of surgery for hepatocellular carcinoma in compensated cirrhosis: a systematic review and meta-analysis. Hepatology.

[86] Llop E., Berzigotti A., Reig (2012). Assessment of portal hypertension by transient elastography in patients with compensated cirrhosis and potentially resectable liver tumors. J Hepatol.

[87] Allaire M., Campion B., Demory A. (2023). Baveno VI and VII criteria are not suitable for screening for large varices or clinically significant portal hypertension in patients with hepatocellular carcinoma. Aliment Pharmacol Ther.

[88] Cheng X., Tang Y., He Q. (2024). Spleen-dedicated stiffness measurement performed well to rule out high-risk varices in HBV-related hepatocellular carcinoma: screening for high-risk varices in HCC. Aliment Pharmacol Ther.

[89] Parikh N.D., Jones P., Salgia R. (2024). Development and validation of a noninvasive model for the detection of high-risk varices in patients with unresectable HCC. Clin Gastroenterol Hepatol : official Clin Pract J Am Gastroenterological Assoc.

[90] Giudicelli H., Andraud M., Wagner M. (2023). Portal-hypertension features are associated with ascites occurrence and survival in patients with hepatocellular carcinoma treated by external radiotherapy. United Eur Gastroenterol J.

[91] Sultanik P., Campani C., Larrey E. (2024). Portal hypertension is associated with poorer outcome and clinical liver decompensation in patients with HCC treated with Atezolizumab-Bevacizumab. Dig Liver Dis.

[92] Pereyra D., Mandorfer M., Santol J. (2024). Von Willebrand factor antigen improves risk stratification for patients with a diagnosis of resectable hepatocellular carcinoma. Ann Surg Oncol.

[93] Sharma P., Mishra S.R., Kumar M. (2012). Liver and spleen stiffness in patients with extrahepatic portal vein obstruction. Radiology.

[94] Elkrief L., Hernandez-Gea V., Senzolo M. (2024). Portal vein thrombosis: diagnosis, management, and endpoints for future clinical studies. Lancet Gastroenterol Hepatol.

[95] Dajti E., Ravaioli F., Colecchia A. (2019). Liver and spleen stiffness measurements for assessment of portal hypertension severity in patients with Budd chiari syndrome. Can J Gastroenterol Hepatol.

[96] De Gottardi A., Sempoux C., Berzigotti A. (2022). Porto-sinusoidal vascular disorder. J Hepatol.

[97] Seijo S., Reverter E., Miquel R. (2012). Role of hepatic vein catheterisation and transient elastography in the diagnosis of idiopathic portal hypertension. Dig Liver Dis.

[98] Elkrief L., Lazareth M., Chevret S. (2021). Liver stiffness by transient elastography to detect porto-sinusoidal vascular liver disease with portal hypertension. Hepatology.

[99] Moga L., Paradis V., Ferreira-Silva J. (2024). Performance of spleen stiffness measurement to rule out high-risk varices in patients with porto-sinusoidal vascular disorder. Hepatology.

[100] Pizzo E., Avsar T.S., Abraldes J.G. (2024 Oct). Cost effectiveness of the Baveno VI criteria compared with endoscopy for high-risk varices in patients with child-pugh A cirrhosis. Clin Gastroenterol Hepatol..

[101] Podrug K., Trkulja V., Zelenika M. (2022). Validation of the new diagnostic criteria for clinically significant portal hypertension by platelets and elastography. Dig Dis Sci.

[102] Dajti E., Ravaioli F., Marasco G. (2022). A combined Baveno VII and spleen stiffness algorithm to improve the noninvasive diagnosis of clinically significant portal hypertension in patients with compensated advanced chronic liver disease. Am J Gastroenterol.

[103] Jindal A., Agarwal S., Sharma S. (2023). Assessment of the performance of non-invasive criteria for the evaluation of clinically significant portal hypertension in patients with compensated advanced chronic liver disease. Dig Dis Sci.

[104] Jachs M., Sandmann L., Hartl L. (2024). Validation of Baveno VII criteria and other non-invasive diagnostic algorithms for clinically significant portal hypertension in hepatitis delta. J Hepatol.

[105] Kim H.Y., So Y.H., Kim W. (2019). Non-invasive response prediction in prophylactic carvedilol therapy for cirrhotic patients with esophageal varices. J Hepatol.

[106] Marasco G., Dajti E., Ravaioli F. (2020). Spleen stiffness measurement for assessing the response to beta-blockers therapy for high-risk esophageal varices patients. Hepatol Int.

[107] Jansen C., Moller P., Meyer C. (2018). Increase in liver stiffness after transjugular intrahepatic portosystemic shunt is associated with inflammation and predicts mortality. Hepatology.

[108] Han H., Yang J., Jin W.K. (2021). Diagnostic value of conventional ultrasound and shear wave elastography in detecting transjugular intrahepatic portosystemic shunt dysfunction. Acta radiologica.

